# Direct in situ labeling of target drugs with a fluorophore probe to improve MALDI-MS detection sensitivity in micro-liter plasma

**DOI:** 10.1038/s41598-019-47147-y

**Published:** 2019-07-25

**Authors:** Yi-Shan Li, Chi-Yu Lu

**Affiliations:** 1 Department of Biochemistry, College of Medicine, Kaohsiung Medical University, Kaohsiung, 80708 Taiwan; 2 Research Center for Environmental Medicine, Kaohsiung Medical University, Kaohsiung, 80708 Taiwan; 3 Institute of Medical Science and Technology, National Sun Yat-sen University, Kaohsiung, 80424 Taiwan; 4 Department of Medical Research, Kaohsiung Medical University Hospital, Kaohsiung, 80708 Taiwan

**Keywords:** Mass spectrometry, Bioanalytical chemistry

## Abstract

Nonsteroidal anti-inflammatory drugs (NSAIDs) are widely used for symptomatic relief from fever, inflammation, and chronic pain associated with a variety of human disorders. Long-term usage of these drugs can result in severe syndromes; hence, their dose should be controlled carefully and their side effects such as Stevens–Johnson syndrome, toxic epidermal necrolysis, phototoxicity, acute interstitial nephritis, gastrointestinal bleeding, cardiovascular diseases, and liver injury should be considered. Furthermore, the widely used combination of NSAIDs as over-the-counter (OTC) drugs with other drugs leads to adverse drug–drug interactions. Therefore, development of a throughput method to rapidly screen 20 NSAIDs in biological samples is necessary to safeguard human health. In this work, we selected a suitable fluorophore probe coupled with *in situ* micro-labeling (<2 min) on stainless plate for the fast detection of NSAIDs in plasma samples at the micro-liter level (5 μL) without complicated sample preparation and separation. Every step undertaken in the protocol was also at the micro-liter level; thus, a small amount of blood collected from the human finger will suffice to determine the drug concentration in blood using the proposed method. Furthermore, the proposed method we developed was also matched the modern trends of green analytical chemistry towards miniaturization of analytical methodologies.

## Introduction

Nonsteroidal anti-inflammatory drugs (NSAIDs) have been used worldwide to provide symptomatic relief from fever, inflammation, and pain associated with a variety of human disorders^1–3^ by inhibiting cyclooxygenases^4,5^. Chronic pain affects approximately 27% of the adult population in Europe and more than 100 million adults in the US^6^. Documented data have shown that more than 30 million people use NSAIDs every day, and these drugs account for 60% of the US over-the-counter (OTC) analgesic market^7,8^. However, NSAIDs have been reported to be the second most common cause of drug-induced hypersensitivity reactions^9,10^. NSAIDs are thought to cause approximately 7000–16500 deaths every year in the US^11^. These severe adverse reactions of NSAIDs, such as Stevens–Johnson syndrome and toxic epidermal necrolysis, should be considered carefully^12–15^.

Recent research has shown that more than 300 drugs (including NSAIDs) have been reported to induce an inflammatory reaction (phototoxicity) or a T-cell-mediated reaction (photoallergy)^16,17^. Other side effects induced by NSAIDs include acute interstitial nephritis (or acute kidney injury)^18–20^, gastrointestinal bleeding^21,22^, cardiovascular diseases^23,24^, liver injury^25,26^, and increase of oxidative stress and apoptosis^27–29^. Furthermore, NSAIDs are widely combined in OTC drugs with other drugs, leading to drug–drug interactions^30,31^. Modern research has attempted to utilize the anti-cancer activity of NSAIDs because cancer is a major cause of death worldwide, with a death toll of 7.6 million or about 13% of all deaths per year^32,33^. Hence, the development of a throughput method to rapidly screen NSAIDs in biological samples may be an effective way to avoid drug-induced adverse reactions.

NSAIDs may be grouped as salicylates, arylalkanoic acids, 2-arylproprionic acids or profens, N-arylanthranilic acids or fenamic acids, pyrazolidine derivates, oxicams, sulfonanilides, and others^34–36^. Many clinicians attempted to understand the relation between the side effects mentioned above and the NSAID concentration in plasma, it is difficult to detect the desired signals without sample preparation and column separation.

Popular sample preparation methods, such as liquid liquid extraction (LLE) and solid phase extraction (SPE), have been widely utilized in biological sample analysis. The disadvantages of LLE and SPE include their labor-intensive and time-consuming aspects, the expenses incurred, the tendency of these techniques to form emulsions, the need for evaporation of large volumes of solvents and disposal of cartridges, use of toxic or flammable chemicals, trial-and-error processes, and the lack of much systematization^37–39^. Chemical derivatization, a time-consuming process, has also proven to be a suitable strategy for assisting in the sample preparation procedures^40,41^. Matrix-assisted laser desorption ionization time-of-flight mass spectrometry (MALDI-TOF MS) can offer many advantages in clinical diagnostic laboratories, such as low acquisition and operating costs, ease of use, ruggedness, and high throughput^42,43^. Before sample detection by MALDI-TOF MS, two simple experimental procedures involve introducing a spot sample and matrix on the target plate and drying for ease of handling. Hence, MALDI-TOF MS is a suitable tool to develop a high-throughput and fast screening method for analysis of NSAIDs.

**Figure 1:**
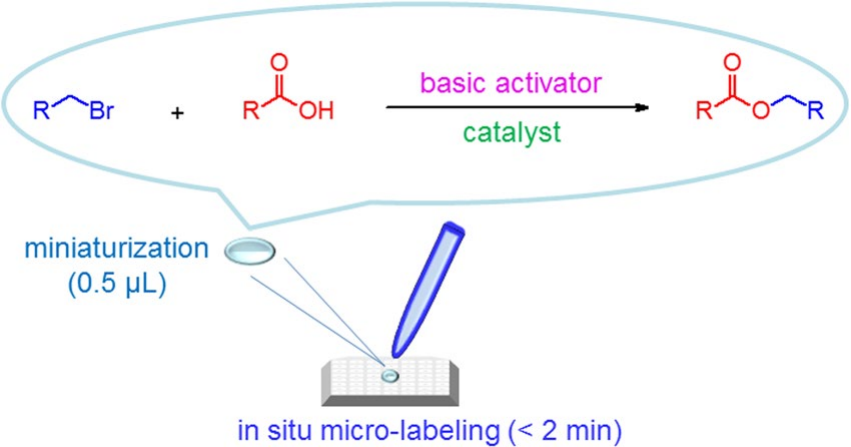
Diagram of micro-labeling of NSAIDs in human plasma.

In this work, *in situ* micro-labeling of NSAIDs with a suitable fluorophore probe was utilized and then the sample solution was spotted on the target plate until dryness. This protocol included the simple sample preparation and chemical labeling steps. This design effectively shortened the derivatization time without extra sample preparation. Several parameters associated with NSAID micro-labeling were studied and optimized. To optimize the micro-labeling efficiency, ibuprofen (a popular OTC drug) was selected as the model analyte because it is the most frequently prescribed NSAID^44^ and it is also associated with male reproductive disorders^45^. All the target analytes in a small amount of human plasma (5 μL) could be detected by MALDI-TOF MS and every step in the protocol was also at the micro-liter or sub- micro-liter level. In summarized, the advantages of the simple sample preparation in the proposed study contain no solvent waste for LLE, no disposable cartridges for SPE, no mobile phase for liquid chromatography, no single-use vails for autosampler, only consumption of micro-liter level solvent for micro-labeling and using a reusable target plate for detection. The reaction scheme for micro-labeling of NSAIDs in this work was shown in Fig. 1.

## Experiment

### Chemicals and reagents

Aspirin, diflunisal, salicylic acid, salsalate, ibuprofen, fenoprofen, flurbiprofen, ketoprofen, oxaprozin, naproxen, loxoprofen, tiaprofenic acid, indometacin, etodolac, aceclofenac, ketorolac, sulindac, diclofenac, mefenamic acid, flufenamic acid, α-cyano-4-hydroxycinnamic acid (CHCA), 2,5-dihydroxybenzoic acid (DHB), 2-mercaptobenzothiazole (MBT), 4-mercaptobenzoic acid (MBA), 18-crown-6, 4′-nitrobenzo-18-crown-6, 2-hydroxymethyl-18-crown-6, benzo-18-crown-6, dicyclohexano-18-crown-6, 4′-aminobenzo-18-crown-6, 2-(bromomethyl)-1,3-benzothiazole (Br-MBT), 8-(bromomethyl) quinoline (Br-MQ), 4-bromomethyl-6,7-dimethoxy coumarin (Br-DMC), 7-acetoxy-4-(bromomethyl) coumarin (Br-MAC), 3-(bromoacetyl) coumarin (Br-AC), 1-(bromoacetyl)pyrene (Br-AP) and 9-(bromomethyl) acridine (Br-MA) were purchased from Sigma-Aldrich (St. Louis, MO, USA). Acetonitrile, potassium carbonate (K_2_CO_3_), potassium bicarbonate (KHCO_3_), potassium hydroxide (KOH), and trifluoroacetic acid were purchased from Merck (Darmstadt, Germany). Ibuprofen-d3 (internal standard, IS) was obtained from Toronto Research Chemicals (Toronto, Canada). All reagents were analytical grade. Deionized water was obtained from a Millipore Milli-Q (Bedford, MA, USA) water purification system.

### Working solutions

All NSAID stock and IS solutions (1 mg/mL) were prepared in acetonitrile. An 18-crown-6 (100 mM) solution was also prepared in acetonitrile. Stock solutions containing Br-MBT, Br-MQ, Br-DMC, Br-MAC, Br-AC, Br-AP, and Br-MA (100 mM) were prepared in acetonitrile. A trifluoroacetic acid solution (0.1%) was prepared in water. The aqueous basic activator (KOH, KHCO_3_, and K_2_CO_3_) stock solutions were prepared as saturated solutions in water. For MALDI-TOF MS analyses, matrices (10 mg/mL) were prepared in 50:50 (v/v) acetonitrile:0.1% trifluoroacetic acid.

### Micro-labeling procedure

Drug-free plasma samples were spiked with different NSAIDs. To build the calibration curves, human plasma (5 μL) spiked with different ibuprofen concentrations (final concentration range of 1–100 μg/mL with ibuprofen-d3 at 35 µg/mL) were transferred into Eppendorf tubes. Calibration curves were built according to the different ibuprofen concentrations (1, 5, 10, 25, 50 and 100 μg/mL). To remove the plasma protein, 10 μL of K_2_CO_3_ (6 M) and 10 μL of 18-crown-6 (5 mM) were added. After centrifugation at 13000 rpm for 10 min, the undesired plasma clot was removed and 10 μL of Br-MBT (10 mM) was added. Then, 0.5 μL of this solution was pipetted onto a stainless target plate and heated at 50 °C for 2 min to drying.

### Instrumentation for MALDI-TOF MS

For NSAID analyses, all the mass spectra were obtained on a MALDI interface with a TOF analyzer (Autoflex III Smartbeam) equipped with a Nd:YAG laser (355 nm) from Bruker Daltonics (Billerica, MA, USA). After adding 0.5 µL of a sample solution on a ground target plate (Bruker Daltonics) and heating it to dry, 0.5 µL of the matrix solution (CHCA) was spotted. Mass spectra were collected in the positive ion reflector mode by summing 2000 laser shots, and the software FlexAnalysis (Bruker Daltonics) was used to process the acquired data.

### Instrumentation for confirming NSAID derivatives

The nano ultra-performance liquid chromatographic system (nanoUPLC) used was manufactured by Waters (Milford, MA, USA). Tandem mass spectrometry was performed with an LTQ Orbitrap Discovery hybrid Fourier transform mass spectrometer (Thermo Fisher Scientific, Inc. Bremen, Germany). The LTQ Orbitrap was operated in the positive ion mode with a nanospray source and at a resolution of 30000. Voltages at the source, tube lens, and capillary were set to 2.3 kV, 80 V, and 28 V, respectively. The spray capillary temperature was set to 200 °C. NSAID derivatives were analyzed with a concentrated column (Symmetry C18, 5 μm, 180 μm × 20 mm) and a nano-flow column (BEH C18, 1.7 μm, 75 μm × 150 mm) purchased from Waters. After micro-labeling, sample solutions (2 μL) were injected and separated at a flow rate of 300 nL/min. Mobile phase A was 0.1% formic acid and mobile phase B was acetonitrile (containing 0.1% formic acid). The gradient conditions were t = 0–1 min, hold B at 1%; t = 1–5 min, increase B from 12 to 100%; t = 5–45 min, hold B at 100%; t = 45–60 min, decrease B from 100 to 1%. NanoUPLC system in this work was only used for identification of NSAID derivatives, not for routine analysis of NSAIDs in plasma samples.

## Results and Discussion

**Figure 2:**
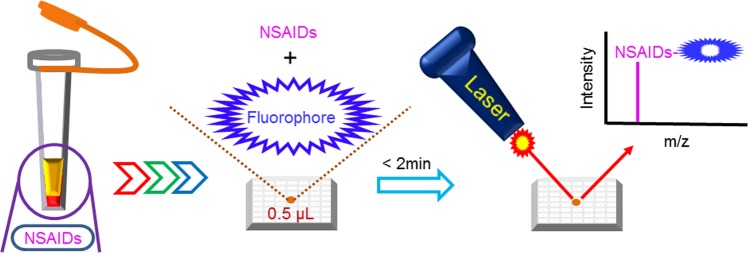
Schematic diagram of the MALDI-TOF MS method utilized to detect NSAIDs in human plasma.

In this study, we established a fast method (using ibuprofen as a test analyte) to detect NSAIDs in human plasma by MALDI-TOF MS. Before the micro-labeling procedure, we attempted to detect ibuprofen (classified as a propionic acid derivative NSAID) in the spiked plasma directly after adding acetonitrile to precipitate the undesired components and then attempting to detect the ibuprofen signal by MALDI-TOF MS. Unfortunately, we could not detect the significant [M + H]^+^ signal of ibuprofen at m/z 207. Hence, detection of ibuprofen in plasma directly was difficult. To enhance the detection sensitivity, selection of a suitable matrix and combination with labeling and an optimized probe were evaluated. Figure 2 shows a schematic diagram of the developed MALDI-TOF MS method for NSAID analysis by labeling with fluorophore probes.

### Matrix and fluorophore probe selection

Matrix selection is the simplest method of choice to change the sensitivity when detecting target compounds by MALDI-TOF MS. Different matrices were used to study the analyte signal after ionization by the MALDI interface, such as CHCA, DHB, MBT, and MBA. The results in Fig. S1 indicate that CHCA was the most suitable matrix to obtain the best response for the detection of ibuprofen derivative in human plasma. Labeling the analyte with a suitable tag is an alternative way to increase the detection sensitivity, and this strategy could be used to produce new side products without suitable extraction and separation. Hence, several fluorophore probes were tested, such as Br-MBT, Br-MQ, Br-DMC, Br-MAC, Br-AC, Br-AP, and Br-MA. These probes could label the carboxyl group of NSAIDs by an alkylating reaction under basic conditions. The results in Table S1 indicate that Br-MBT (which contains a benzothiazole fluorophore) was the most suitable reagent to label ibuprofen. MBT (which also contains a benzothiazole fluorophore) is a kind of matrix used in the MALDI interface^46^ and therefore an analyte labeled with this aromatic ring could undergo enhanced detection sensitivity.

### Optimization of micro-labeling procedure

**Figure 3:**
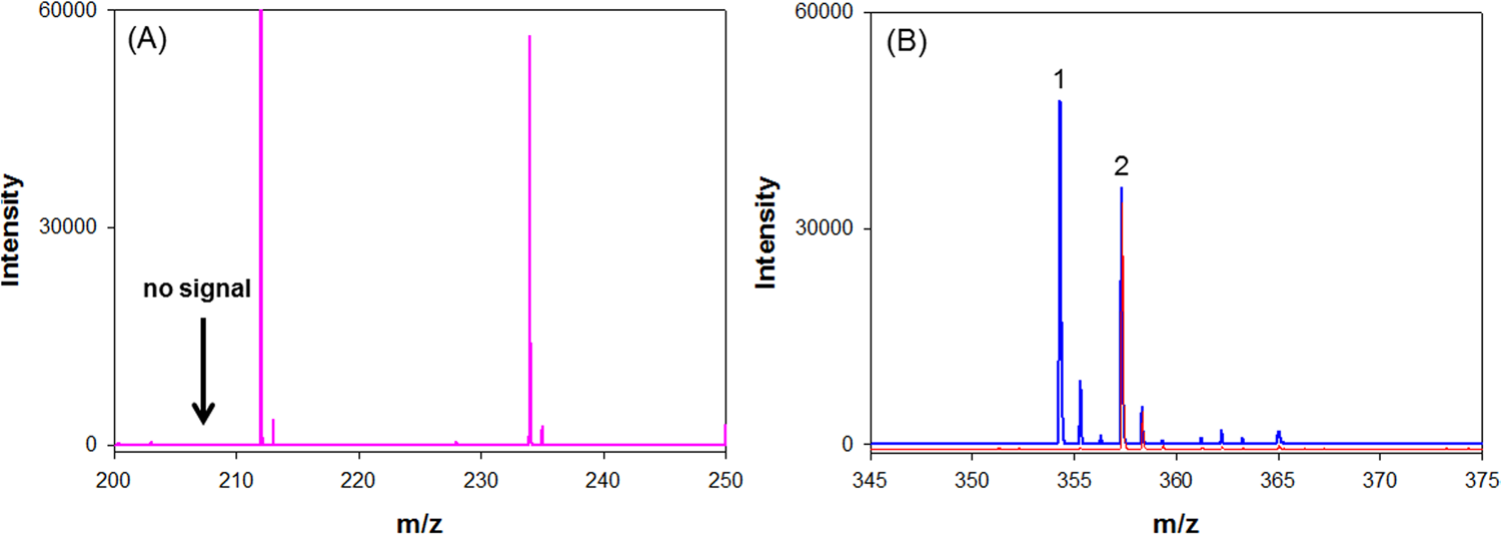
Typical mass spectra for (**A**) the analysis of ibuprofen spiked in human (100 μg/mL) plasma after adding acetonitrile to precipitate the undesired components and [M + H]^+^ signal of ibuprofen at m/z 207 could not be detectable; (**B**) the analysis of ibuprofen in human plasma under the optimized micro-labeling conditions. [M + H]^+^ signals of ibuprofen (peak 1) and ibuprofen-d3 (peak 2) derivatives appear at m/z 354 and 357, respectively. The red line is due to the plasma blank and blue line is attributable to the plasma spiked with 100 and 35 μg/mL of ibuprofen and ibuprofen-d3, respectively.

To develop the suitable micro-labeling steps, many factors associated with the detection of ibuprofen by MALDI-TOF MS were tested. To identify an optimal Br-MBT concentration for the detection of the ibuprofen derivative, different concentrations of Br-MBT (0.5–20 mM) were tested. The results in Fig. S2 indicate that a Br-MBT concentration of 10 mM was the most suitable for determining the ibuprofen derivative. For the micro-labeling process, the carboxylic acid of ibuprofen dissociated and drove an alkylation reaction by Br-MBT under basic conditions. To study the effects of basic activators, different potassium salts (KOH, KHCO_3_, and K_2_CO_3_) were tested. The results in Fig. S3 indicate that K_2_CO_3_ was the most suitable for detection of the ibuprofen derivative. To study the effects of the concentration of K_2_CO_3_, different concentrations of K_2_CO_3_ (1 M to saturated) were tested. The results in Fig. S4 indicate that 6 M of K_2_CO_3_ was suitable for detection of the ibuprofen derivative. A high concentration of K_2_CO_3_ could suppress the target signal of the analyte; thus, the detection signal could be enhanced by adding crown ether. Crown ethers have many ether groups that they can use as ligands for capturing some metal cations. For example, 18-crown-6 has six oxygen atoms with high affinity to bind potassium cations to produce stable complexes. To study the effects of the crown ether used, different ethers (18-crown-6, 4′-nitrobenzo-18-crown-6, 2-hydroxymethyl-18-crown-6, benzo-18-crown-6, dicyclohexano-18-crown-6, and 4′-aminobenzo-18-crown-6) were tested. The results in Fig. S5 indicate that 18-crwon-6 ether without modification was the most suitable for detection of the ibuprofen derivative. 2-Hydroxymethyl-18-crown-6 and 4′-nitrobenzo-18-crown-6 were the second and third most suitable ligand for the purpose. To study the effect of the concentration of 18-crown-6, different concentrations 18-crown-6 (0.1–10 mM) were tested. The results shown in Fig. S6 indicate that 5 mM of 18-crown-6 was suitable for detection of the ibuprofen derivative. To study the effects of the reaction temperature, the reaction was undertaken at different temperatures (30, 50, and 70 °C). The results in Fig. S7 indicate that 50 °C was a suitable reaction temperature for detection of the ibuprofen derivative. Typical mass spectra for the optimized micro-labeling conditions for the analysis of ibuprofen in human plasma with and without derivatization with fluorophore probe are shown in Fig. 3. Without micro-labeling procedure, we could not detect the [M + H]^+^ signal of ibuprofen.

### Analysis of ibuprofen in human plasma

**Figure 4:**
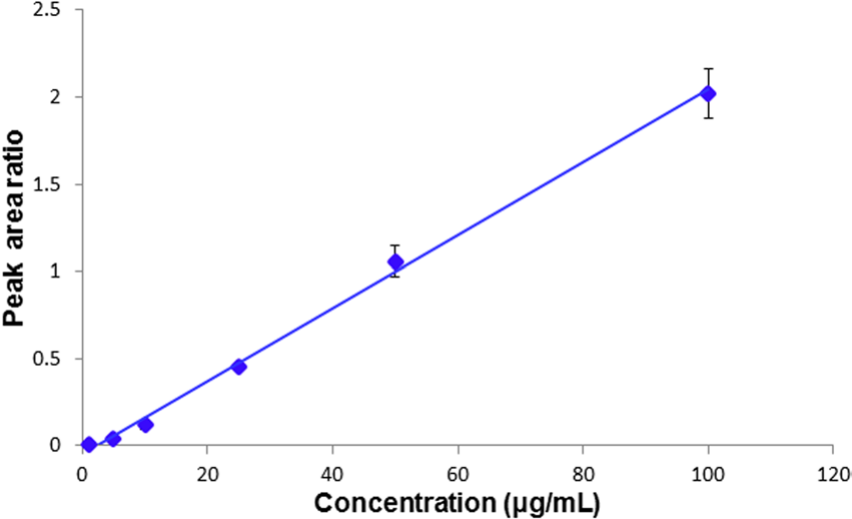
Calibration curves for analysis of ibuprofen spiked in human plasma under the optimized micro-labeling conditions.

For the suitable micro-labeling procedure, the linear range for the detection of ibuprofen was 1–100 μg/mL for plasma at the micro-liter level. The results showed a high linearity with y = (0.0209 ± 0.0015)x–(0.0464 ± 0.0127) and a coefficient of determination (r^2^) = 0.996 (n = 5). Calibration curves were shown in Fig. 4. The recovery was 90–106% and the limit of detection was 0.1 μg/mL. The precision and accuracy of intra- and inter-day analyses of ibuprofen in human plasma were tested at three concentrations (2.5, 20, and 80 μg/mL). Table 1 shows that the RSD and RE were below 11.0% for intra-day (n = 5) and inter-day (n = 5) assays.

**Table 1:** Precision and accuracy of analyses of ibuprofen in human plasma.

Concentration known (μg/mL)	Concentration found (μg/mL)	RSD^a^ (%)	RE^b^ (%)
Intra-day (n = 5)			
80	75.43 ± 1.21	1.6	−5.7
20	21.14 ± 1.20	5.7	+5.7
2.5	2.63 ± 0.06	2.3	+5.2
Inter-day (n = 5)			
80	82.71 ± 2.92	3.5	+3.4
20	17.89 ± 1.09	6.1	−10.6
2.5	2.62 ± 0.11	4.2	+4.8

^a^Relative standard deviation (RSD) = (SD/mean) × 100.

^b^Relative error (RE) = (Concentration found − Concentration known/Concentration known) × 100.

The proposed method under optimized protocol was further utilized to detect plasma ibuprofen in a healthy volunteer by MALDI-TOF MS. Plasma samples were obtained before and after a single oral dose of ibuprofen tablet (400 mg) by utilizing single-use lancets (BD, Franklin Lakes, USA) to prick the finger of the volunteer and then collecting the blood sample in a Microvette capillary blood collection tube (Sarstedt, Nümbrecht, Germany). Only a small amount of plasma sample was collected without using a typical medical syringe. After 2 h, the peak plasma concentration of ibuprofen was 38.76 ± 2.51 µg/mL, consistent with the pharmacokinetics of ibuprofen reported in literature^47^. These results show that the proposed microscale method can be utilized for monitoring ibuprofen in even 5 µL of human plasma. A suitable fluorophore probe Br-MBT could help enhance the ionization and increase the detection sensitivity of ibuprofen in complicated biological samples. Table S2 compares the proposed method in this study and other methods reported in the literature for analysis of ibuprofen in human plasma.

### Stability and selectivity

In order to examine ibuprofen stability in plasma, the peak area ratio of the stability of the ibuprofen derivative to that of the IS was evaluated. Plasma samples were stored at −20 °C and the stability of the ibuprofen derivative, relative to that of IS, was examined over 14 days. No obvious change in ibuprofen derivative/IS ratio was observed, indicating that the ibuprofen derivative is sufficiently stable for MALDI-TOF MS analysis.

In order to examine ibuprofen selectivity in plasma, NSAIDs of different groups (listed in Table 2) were spiked into plasma and then the selectivity of the proposed method under the optimized micro-labeling conditions was tested. NSAIDs such as salicylic acid derivatives (aspirin, diflunisal, salicylic acid, and salsalate), propionic acid derivatives (ibuprofen, naproxen, fenoprofen, ketoprofen, flurbiprofen, oxaprozin, loxoprofen, and tiaprofenic acid), acetic acid derivatives (indomethacin, sulindac, etodolac, ketorolac, diclofenac, and aceclofenac) and anthranilic acid derivatives (mefenamic acid and flufenamic acid) were tested by this proposed method under suitable micro-labeling conditions. The results displayed in Table 2 indicate that all of these tested NSAIDs in human plasma could be detected in microliters of plasma using our proposed MALDI-TOF MS method. All the mass spectra of these NSAID derivatives were displayed in Figs S8-1 to S8-19. To confirm that the micro-labeling protocol was sufficient, the exact mass of these NSAID derivatives were also determined by LTQ Orbitrap. All the molecular weight differences between the theoretical and detected [M + H]^+^ for these NSAID derivatives were below 5 ppm.

**Table 2:** NSAID derivatives confirmed by nanoUPLC-MS/MS.

Classification	Molar weight after labeling ([M + H]^+^)		ppm
	Known	Found	
Salicylic acid derivatives			
aspirin	328.0638	328.0643	1.5
salicylic acid	286.0532	286.0531	−0.3
salsalate	406.0744	406.0748	1.0
diflunisal	398.0657	398.0653	−1.0
Propionic acid derivatives			
ibuprofen	354.1522	354.1539	4.8
fenoprofen	390.1158	390.1166	2.1
flurbiprofen	392.1115	392.1116	0.3
ketoprofen	402.1158	402.1172	3.5
oxaprozin	441.1267	441.1276	2.0
naproxen	378.1158	378.1162	1.1
loxoprofen	394.1471	394.1484	3.3
tiaprofenic acid	408.0723	408.0729	1.5
Acetic acid derivatives			
etodolac	435.1737	435.1758	4.8
aceclofenac	501.0437	501.0447	2.0
ketorolac	403.1111	403.1116	1.2
sulindac	504.1098	504.1092	−1.2
diclofenac	443.0382	443.0395	2.9
indometacin	505.0983	505.0998	3.0
Anthranilic acid derivatives			
mefenamic acid	389.1318	389.1324	1.5
flufenamic acid	429.0879	429.0882	0.7

The annual cost of pain medication used to treat chronic pain associated with NSAIDs is approximately $1.9 billion in the United States^48^. Hence, in this work, we developed an *in situ* micro-labeling method for fast detection of NSAIDs in human plasma by MALDI TOF MS after micro-labeling of a benzothiazole ring to enhance the detection sensitivity. Previous reports stated that 2-mercaptobenzothiazole is a suitable matrix for use in MALDI-TOF MS^46^. The fluorophore Br-MBT contains a benzothiazole ring and a bromomethyl group. The bromomethyl group could react with the carboxylic acids of NSAIDs and then the NSAID derivatives would contain the benzothiazole moiety. Hence, we proposed that the benzothiazole ring attached to the NSAID derivatives could enhance the ionization of NSAIDs in the MALDI interface. Even in complicated biological samples (such as plasma), NSAIDs labeled with the benzothiazole ring could show increased detection sensitivity without column separation. This phenomenon could probably explain why the other fluorophore (such as quinoline, coumarin, pyrene, and acridine) probes tested in this study could not attain the improved detection sensitivity of NSAIDs in MALDI-TOF MS.

For the beginning of Precision Medicine Initiative in 2015^49^, there is a huge demand for novel, robust, and cost-effective diagnostic and prognostic assays^50^. Because of the increasing research interest in personalized medicine, using biomedical devices to deliver tailored diagnostics and therapeutics according to the individual patient becomes very important^51^. Analytical chemistry plays an important role in the area for the detection of target compounds from biological samples. The recent trends of analytical chemistry towards miniaturization of analytical systems and sample preparation methodologies that has resulted in the development of effective and low-cost microextraction techniques^52–54^. These strategies proposed to require smaller amounts of samples, to reduce the consumption of organic solvents from milliliters to just a few microliters and to remove additional cleaning steps^55–57^. For green methods, miniaturization is a clear trend in analytical chemistry motivated by the need to solve the limitations of conventional analytical systems and aimed at providing more efficient and environmentally friendly systems^58,59^. The column separation free strategy and *in situ* micro-labeling procedure of the proposed method under miniaturized scale was an alternative approach towards the green analytical chemistry.

## Conclusion

In this study, we selected a fluorophore probe to directly label NSAIDs in plasma samples without column separation to enhance their detection sensitivity in MALDI-TOF MS. In the strategy proposed in this work, sample separation and purification by an extra chromatographic method are unnecessary after the *in situ* micro-labeling of NSAIDs on the stainless target plate in the micro-scale process. Application of the proposed method to detect NSAIDs in biological samples (such as plasma obtained from finger) by MALDI-TOF MS proved efficient. This high-throughput method could be suitably modified for automation programs to develop an ultra-high throughput instrument in future for detection of NSAIDs in biological samples, which could be very useful in the prevention of NSAID abuse or addiction.

### Compliance with ethical standards

The experiments were approved by the Institutional Review Board of Kaohsiung Medical University Chung-Ho Memorial Hospital. The method was performed in accordance with the approved guidelines and written informed consent was obtained from all participants.

## Supplementary information

Electronic Supplementary Material
