# Supplementary material for: Direct in situ labeling of target drugs with a fluorophore probe to improve MALDI-MS detection sensitivity in micro-liter plasma
Source: Sci Rep. 2019 Jul 25;9:10787. doi: 10.1038/s41598-019-47147-y (PMC6658545; doi:10.1038/s41598-019-47147-y)
Supplement: Supplementary file 1 — Electronic Supplementary Material [file 41598_2019_47147_MOESM1_ESM.pdf]

Electronic Supplementary Material

Direct in situ labeling of target drugs with a fluorophore probe to improve MALDI-MS detection sensitivity in micro-liter plasma

Yi-Shan Li [1] • Chi-Yu Lu [1, 2, 3, 4]

[1] Department of Biochemistry, College of Medicine, Kaohsiung Medical University, Kaohsiung 80708, Taiwan

[2] Research Center for Environmental Medicine, Kaohsiung Medical University, Kaohsiung 80708, Taiwan

[3] Institute of Medical Science and Technology, National Sun Yat-sen University, Kaohsiung 80424, Taiwan

[4] Department of Medical Research, Kaohsiung Medical University Hospital, Kaohsiung 80708, Taiwan

✉ Chi-Yu Lu

cylu@kmu.edu.tw

Table S1. Effects of fluorophore probes on the formation of ibuprofen derivative

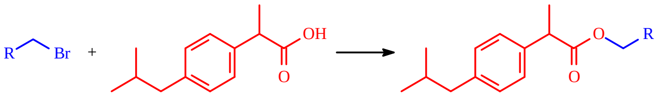

Probe	Structure	Relative response (%)
Br-MBT	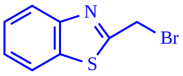	100
Br-MQ	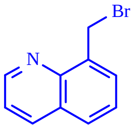	6
Br-DMC	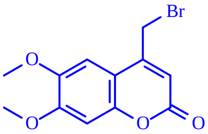	< 1
Br-MAC	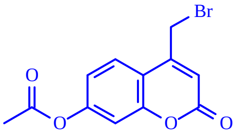	< 1
Br-MA	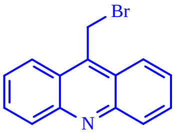	< 1
Br-AC	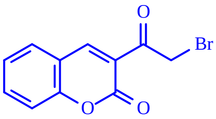	no signal
Br-AP	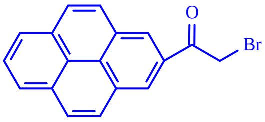	no signal

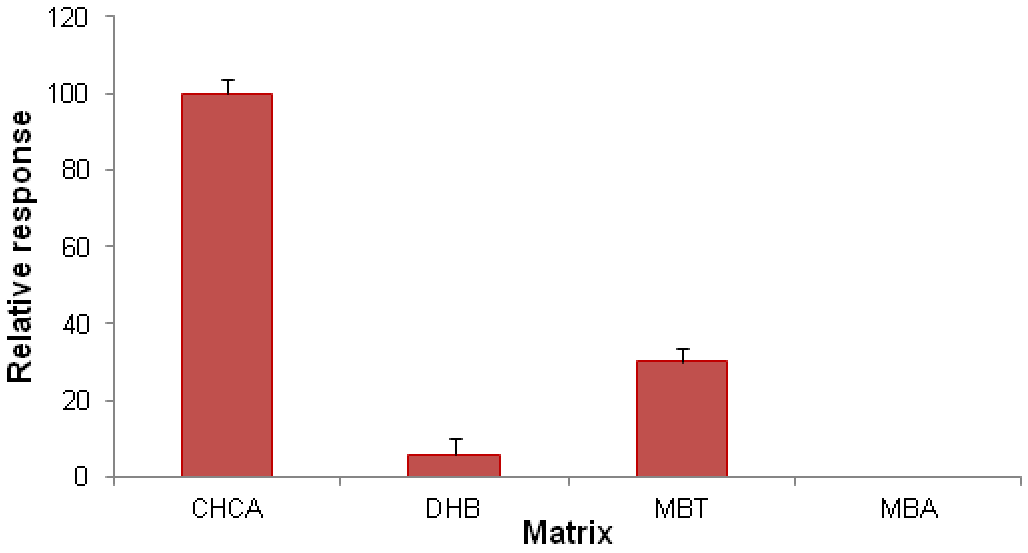

Figure S1 Effects of the matrix on detection of ibuprofen derivative signal in human plasma.

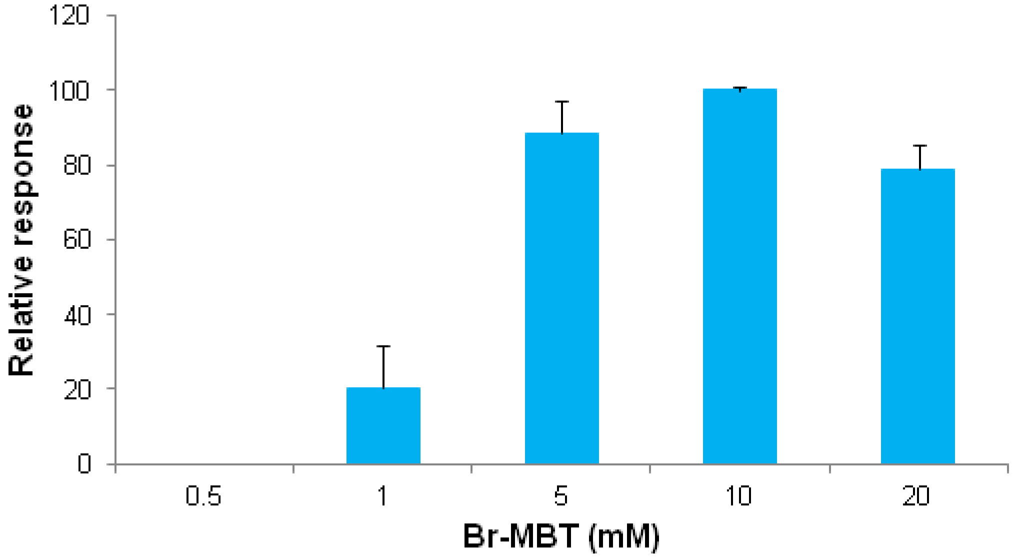

Figure S2 Effect of Br-MBT concentration on formation of the ibuprofen derivative.

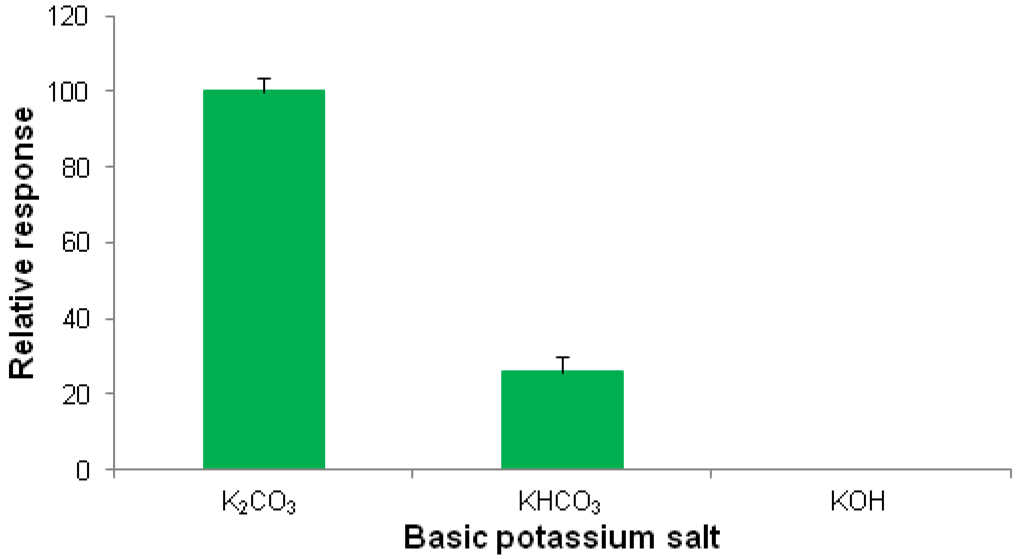

Figure S3 Effects of basic potassium salts on the formation of the ibuprofen derivative.

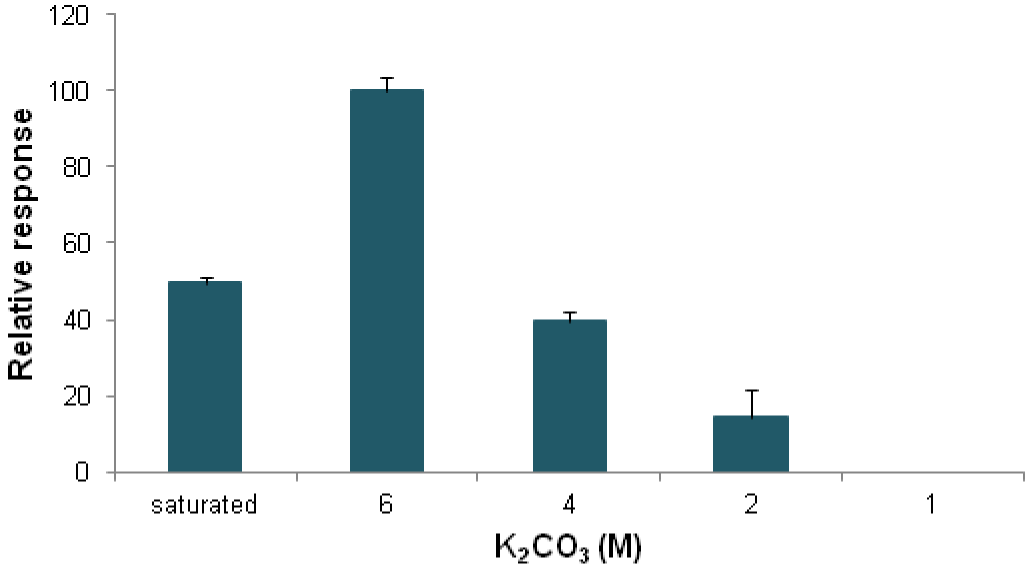

Figure S4 Effects of K_2CO_3 concentration on the formation of the ibuprofen derivative.

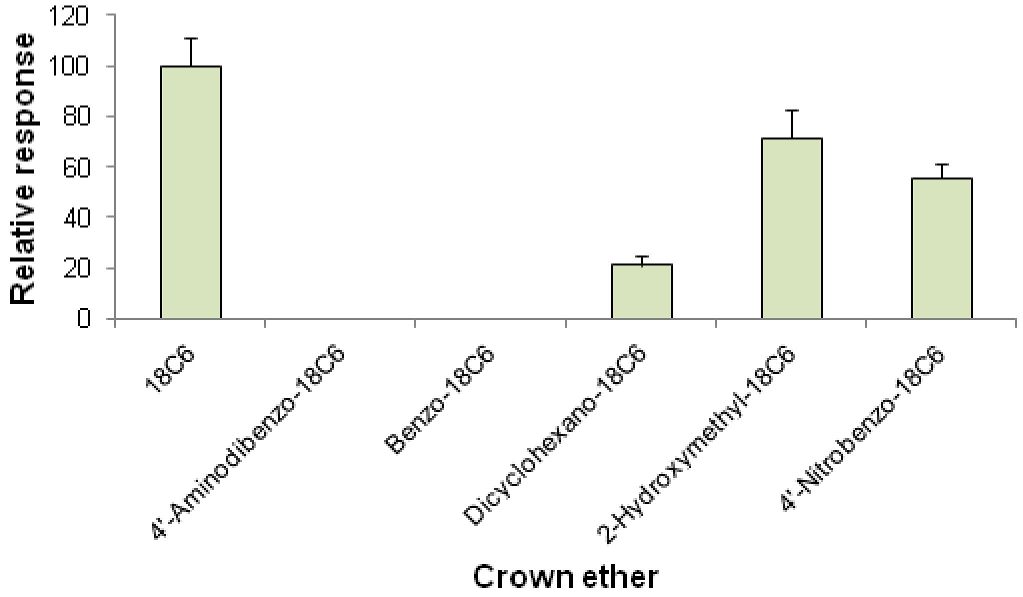

Figure S5 Effect of the different 18-crown-6 (18C6) crown ethers on the formation of the ibuprofen derivative.

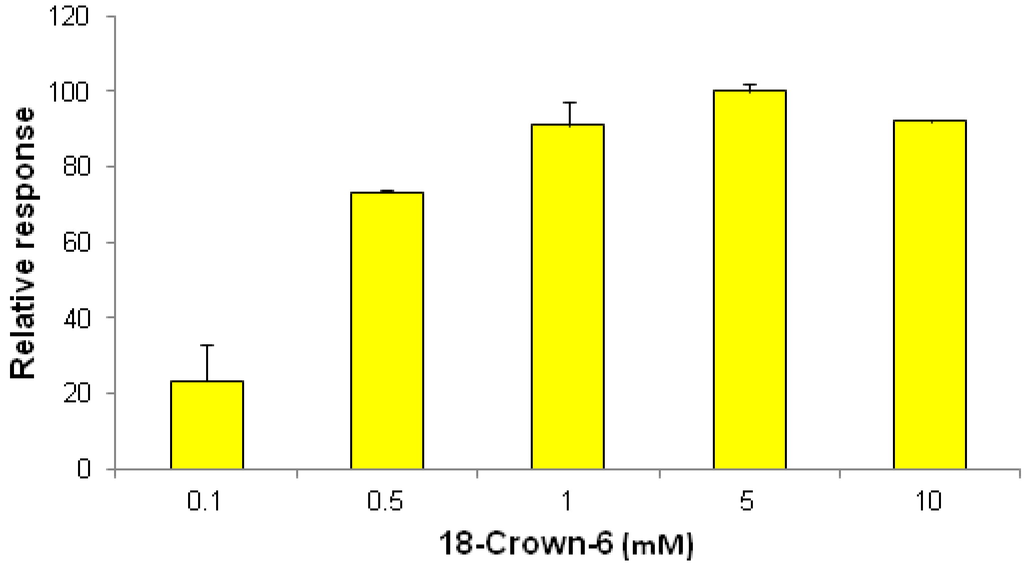

Figure S6 Effect of 18-crown-6 concentration on the formation of the ibuprofen derivative.

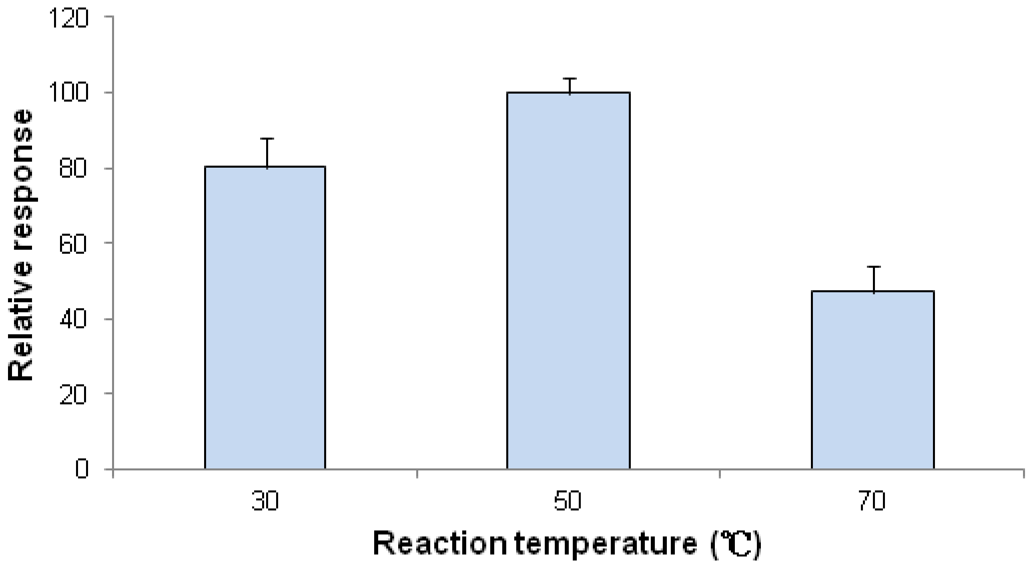

Figure S7 Effect of the reaction temperature on the formation of the ibuprofen derivative.

Table S2. Comparison of current methods for analysis of ibuprofen

Analytical method	Sample	Sample volume (μL)	Sample preparation	Analytical time (min)	LOD (μg/mL)	Mobile phase/ carrier gas	Ref.
LC-UV	human plasma	1000	LLE	3.5	0.04	acetonitrile, phosphoric acid	[1]
LC-FLD	human plasma	500	LLE	20	0.1	acetonitrile, acetic acid, triethylamine	[2]
LC-UV	human plasma	50	SPE	5	-	acetonitrile, phosphoric acid	[3]
LC-UV	human plasma	100	protein precipitation	12.5	1	methanol, potassium hydrogen phosphate	[4]
LC-MS/MS	human plasma	100	LLE	1.5	-	methanol, acetonitrile, ammonium formate buffer	[5]
LC-MS/MS	human plasma	50	LLE SPE	13	0.02	methanol, acetic acid	[6]
LC-MS/MS	human plasma	20	LLE	8	0.02	methanol, formic acid	[7]
LC-UV	human plasma	1000	direct injection	14	0.07	sodium dodecyl sulfate, n-propanol, triethylamine, orthophosphoric acid	[8]
GC-MS/MS	human plasma	10	LLE	8	-	helium	[9]
MALDI-TOF MS	human plasma	5	micro-labeling	< 1	0.1	-	This work

Abbreviations: UV, ultraviolet; FLD, fluorescence detector; MS, mass spectrometry; LC, liquid chromatography; GC, gas chromatography; LLE, liquid-liquid extraction; SPE, solid phase extraction; LOD, limit of detection; Ref., reference.

References

1. Litowitz, H., Olanoff, L. & Hoppel, C.L. Determination of ibuprofen in human plasma by high-performance liquid chromatography. *J. Chromatogr. B Analyt Technol.*

Biomed. Life Sci. **311**, 443-448 (1984).

2. Canaparo, R., Muntoni, E., Zara, G.P., Pepa, C.D., Berno, E., Costa, M. & Eandi, M. Determination of Ibuprofen in human plasma by high-performance liquid chromatography: validation and application in pharmacokinetic study. *Biomed. Chromatogr*. **14**, 219-226 (2000).
3. Farrar, H., Letzig, L. & Gill, M. Validation of a liquid chromatographic method for the determination of ibuprofen in human plasma. *J. Chromatogr. B* **780**, 341-348 (2002).
4. Ganesan, M., Rauthan, K.S., Pandey, Y. & Tripathi, P. Determination of ibuprofen in human plasma with minimal sample pretreatment. *Int. J. Pharm. Sci. Res*. **1**, 120-127 (2010).
5. Seelam, R.R., Chandiran, I.S., Jayaveera, K.N. & Divi, K.R. Quantification of Ibuprofen in human plasma by using high throughput liquid chromatography–tandem mass spectrometric method and its applications in pharmacokinetics. *Arch. Apll. Sci. Res*. **2**, 101-111 (2010).
6. Nakov, N., Petkovska, R., Ugrinova, L., Kavrakovski, Z., Dimitrovska, A. & Svinarov, D. Critical development by design of a rugged HPLC-MS/MS method fordirect determination of ibuprofen enantiomers in human plasma. *J. Chromatogr. B* **992**, 67-75 (2015).
7. Chen, T., Li, Q., Lu, J., Yu, C., Chen, C. & Li, Z. Determination of ibuprofen enantiomers in human plasma by HPLC–MS/MS: validation and application in neonates. *Bioanalysis* **8**, 1237-1250 (2016).
8. Talaat, W. Bioanalytical method for the estimation of co-administered esomeprazole, leflunomide and ibuprofen in human plasma and in pharmaceutical dosage forms using micellar liquid chromatography. *Biomed. Chromatogr*. **31**, e3865 (2017).
9. Tsikas, D., Kayacelebi, A.A., Hanff, E., Mitschke, A., Beckmann, B., Tillmann, H.C., Gutzki, F.M., Müller, M. & Bernasconi, C. GC-MS and GC-MS/MS measurement of ibuprofen in 10-µL aliquots of human plasma and mice serum using [α-methylo-$^{2}H_3$]ibuprofen after ethyl acetate extraction and pentafluorobenzyl bromide derivatization: Discovery of a collision energy-dependent H/D isotope effect and pharmacokinetic application to inhaled ibuprofen-arginine in mice. *J. Chromatogr. B* **1043**, 158-166 (2017).

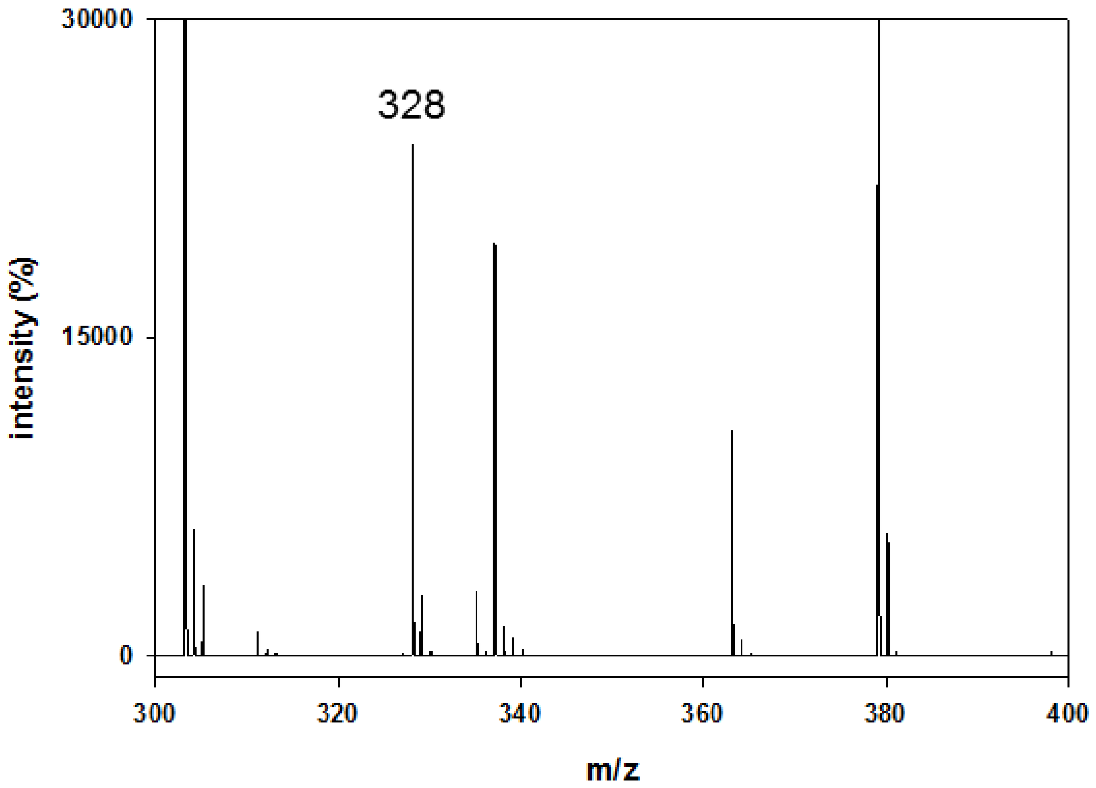

Figure S8-1 Mass spectrum for the analysis of aspirin spiked in human plasma, $[M+H]^+$ signal appears at m/z 328.

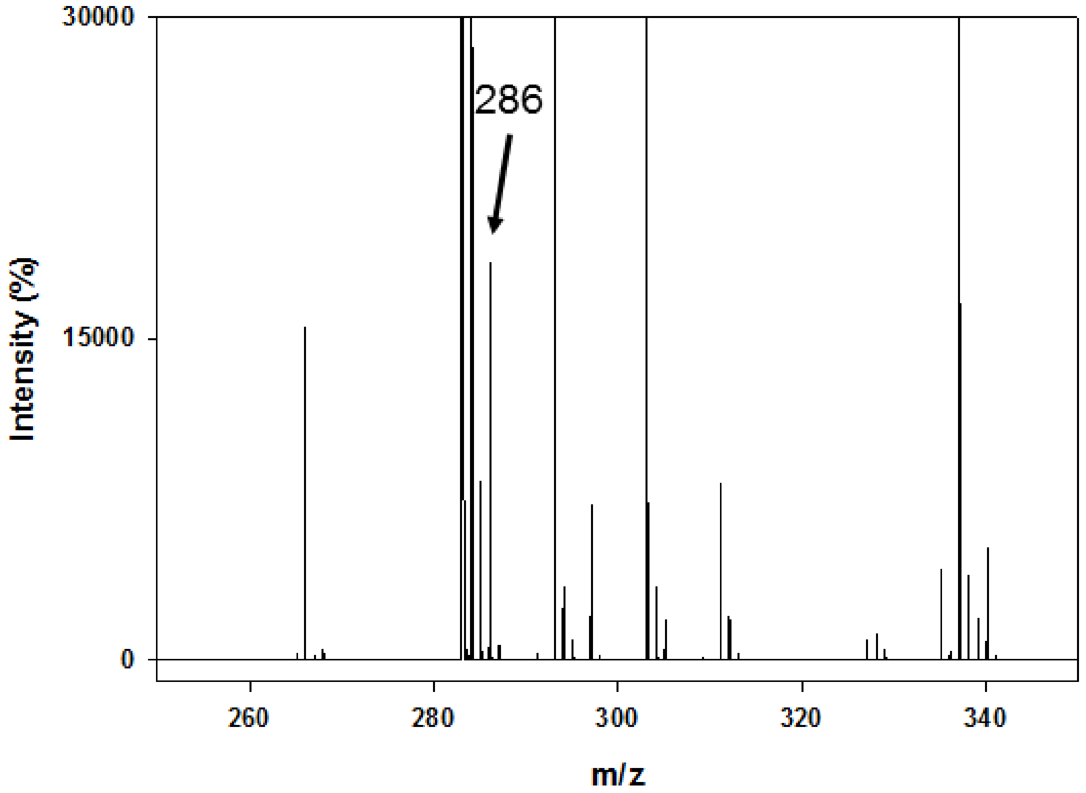

Figure S8-2 Mass spectrum for the analysis of salicylic acid spiked in human plasma, $[M+H]^+$ signal appears at m/z 286.

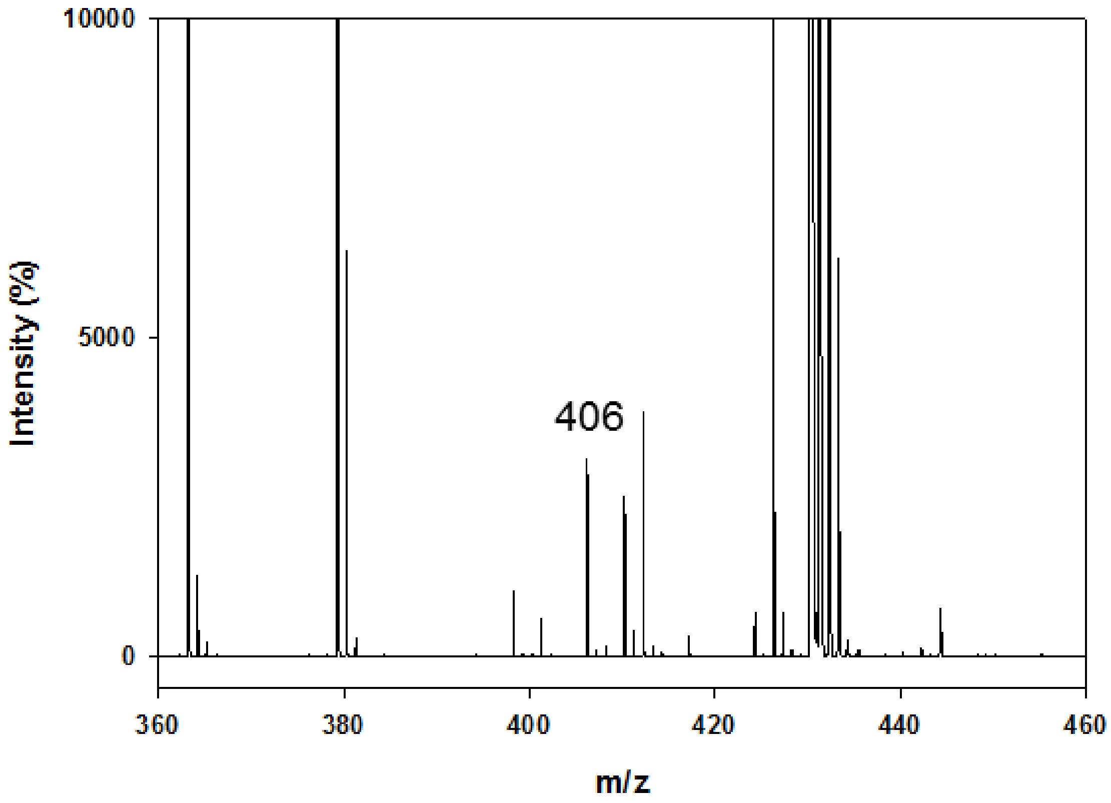

Figure S8-3 Mass spectrum for the analysis of salsalate spiked in human plasma, $[M+H]^+$ signal appears at m/z 406.

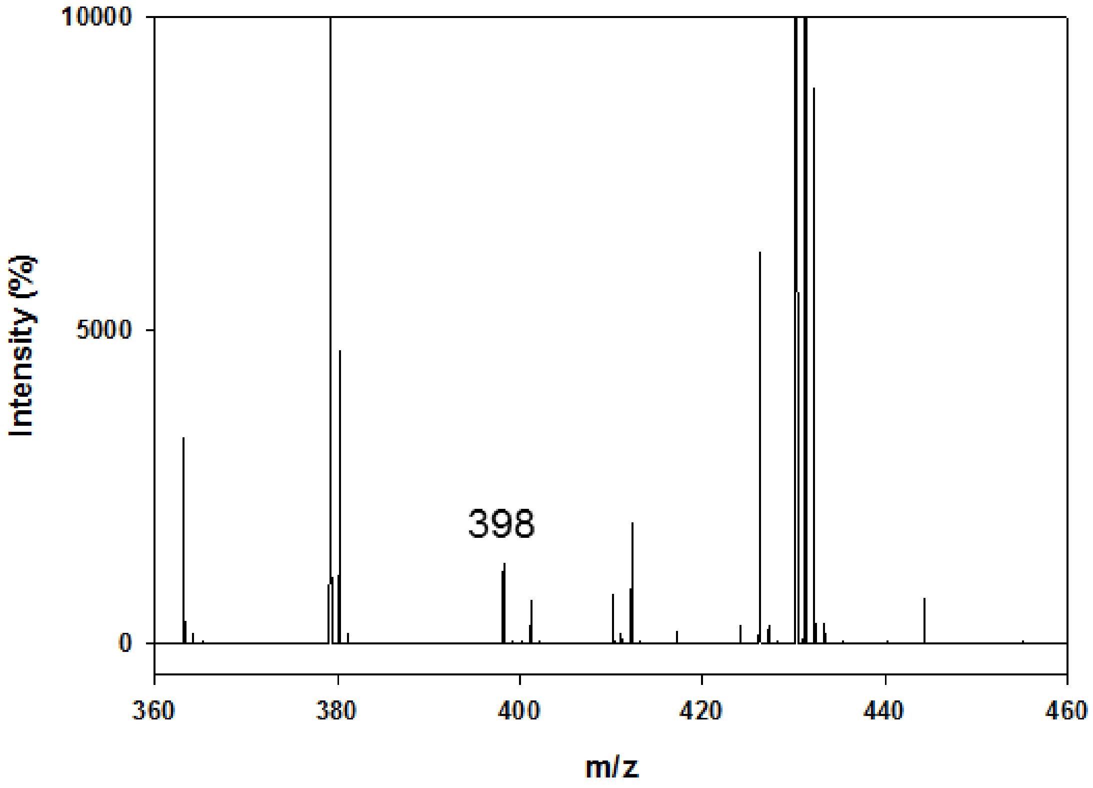

Figure S8-4 Mass spectrum for the analysis of diflunisal spiked in human plasma, $[M+H]^+$ signal appears at m/z 398.

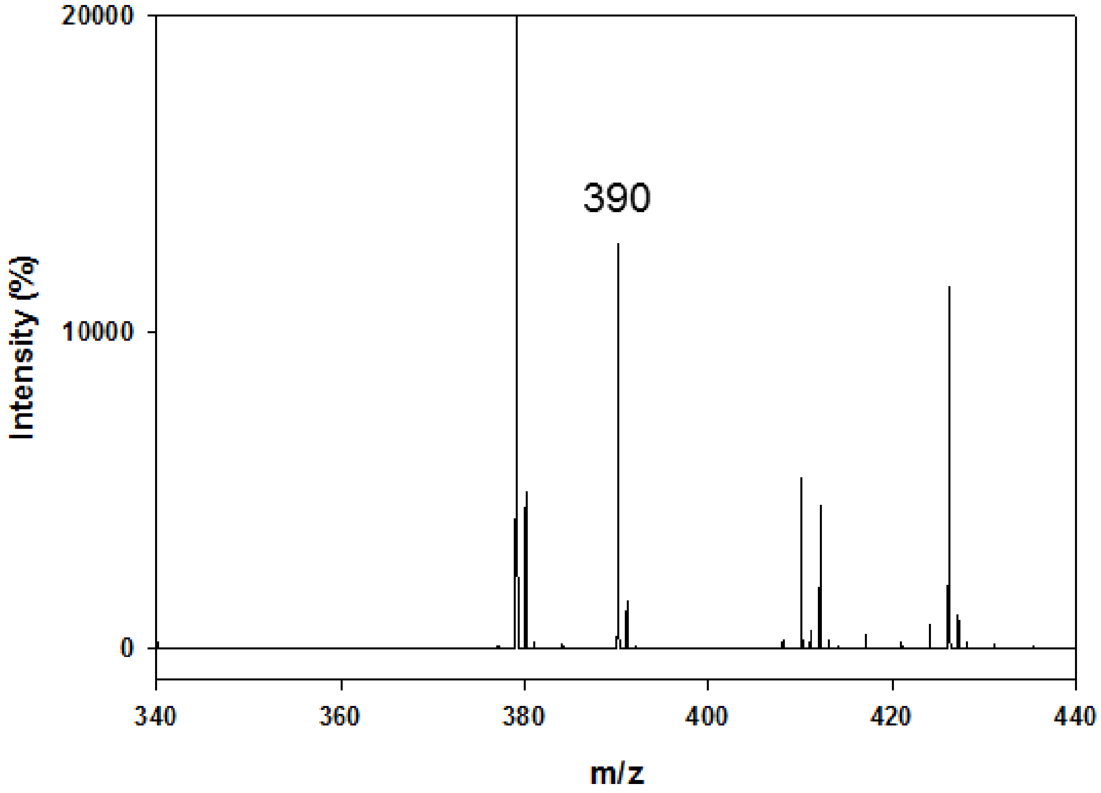

Figure S8-5 Mass spectrum for the analysis of fenoprofen spiked in human plasma, $[M+H]^+$ signal appears at m/z 390.

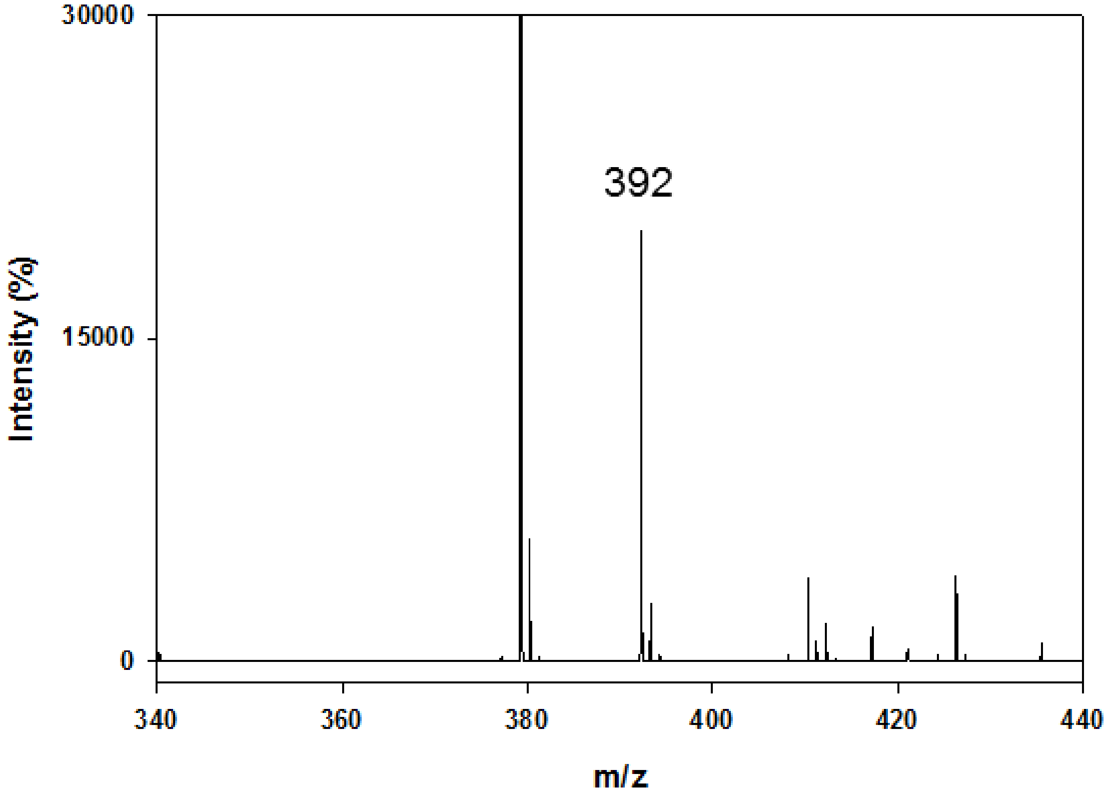

Figure S8-6 Mass spectrum for the analysis of flurbiprofen spiked in human plasma, $[M+H]^+$ signal appears at m/z 392.

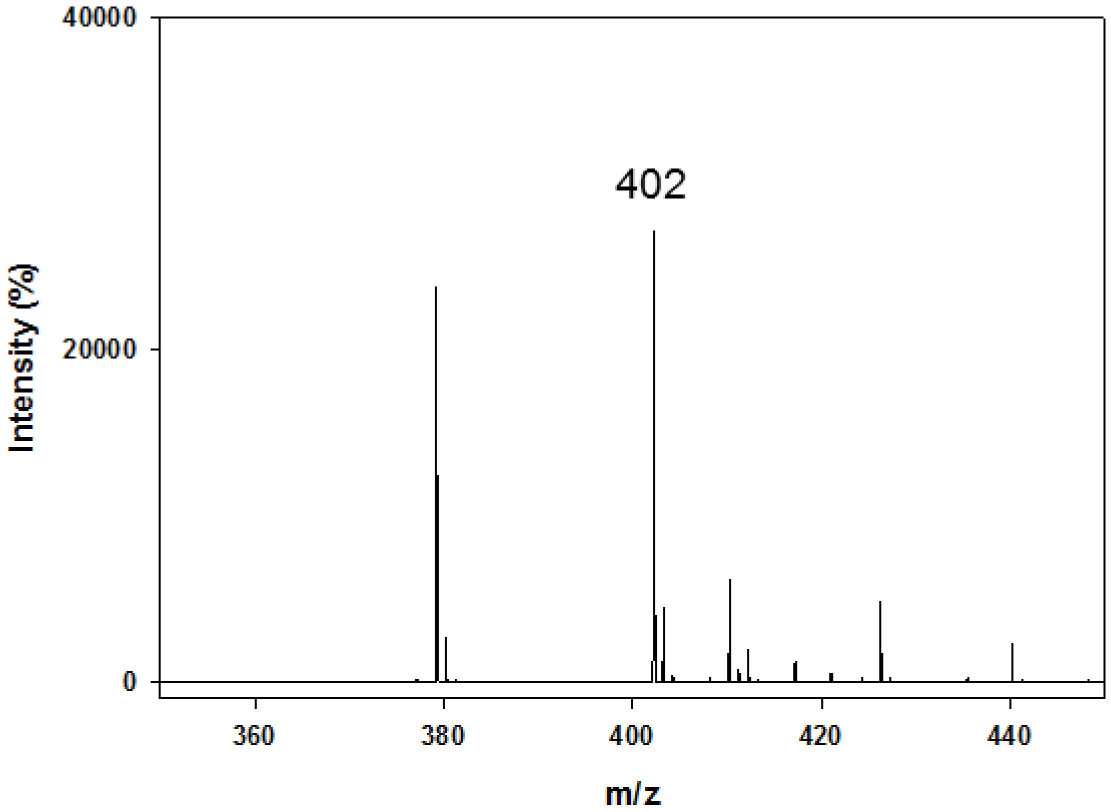

Figure S8-7 Mass spectrum for the analysis of ketoprofen spiked in human plasma, $[M+H]^+$ signal appears at m/z 402.

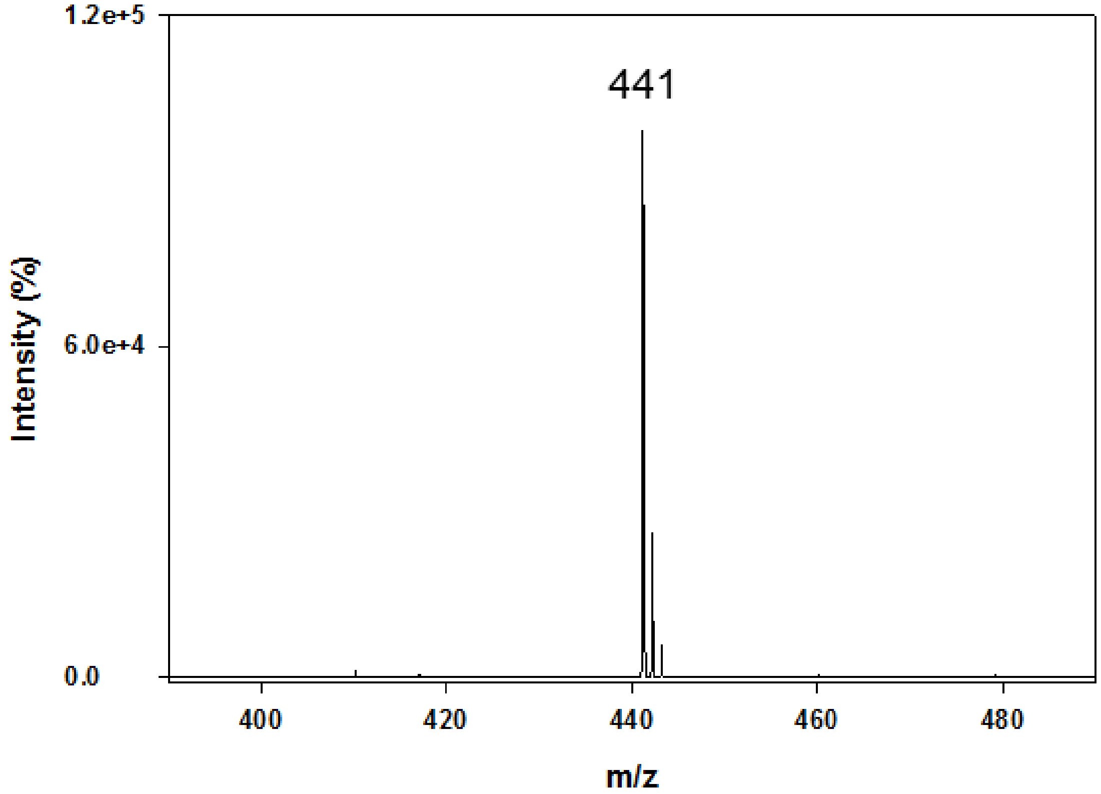

Figure S8-8 Mass spectrum for the analysis of oxaprozin spiked in human plasma, $[M+H]^+$ signal appears at m/z 441.

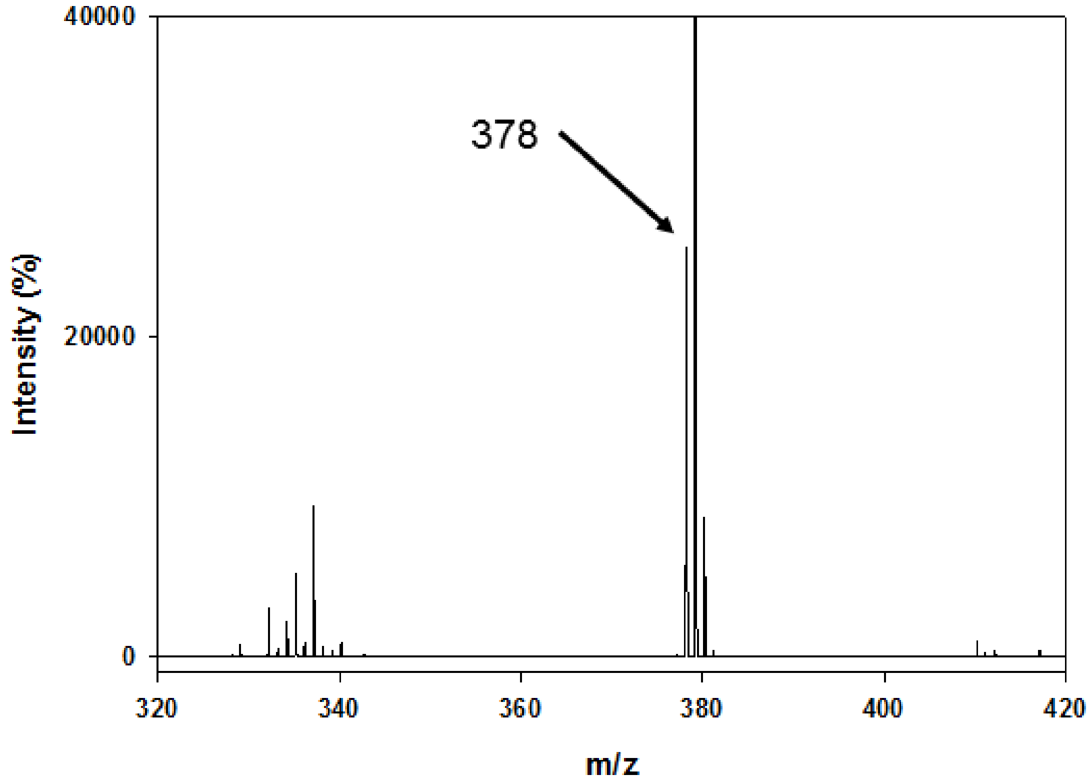

Figure S8-9 Mass spectrum for the analysis of naproxen spiked in human plasma, $[M+H]^+$ signal appears at m/z 378.

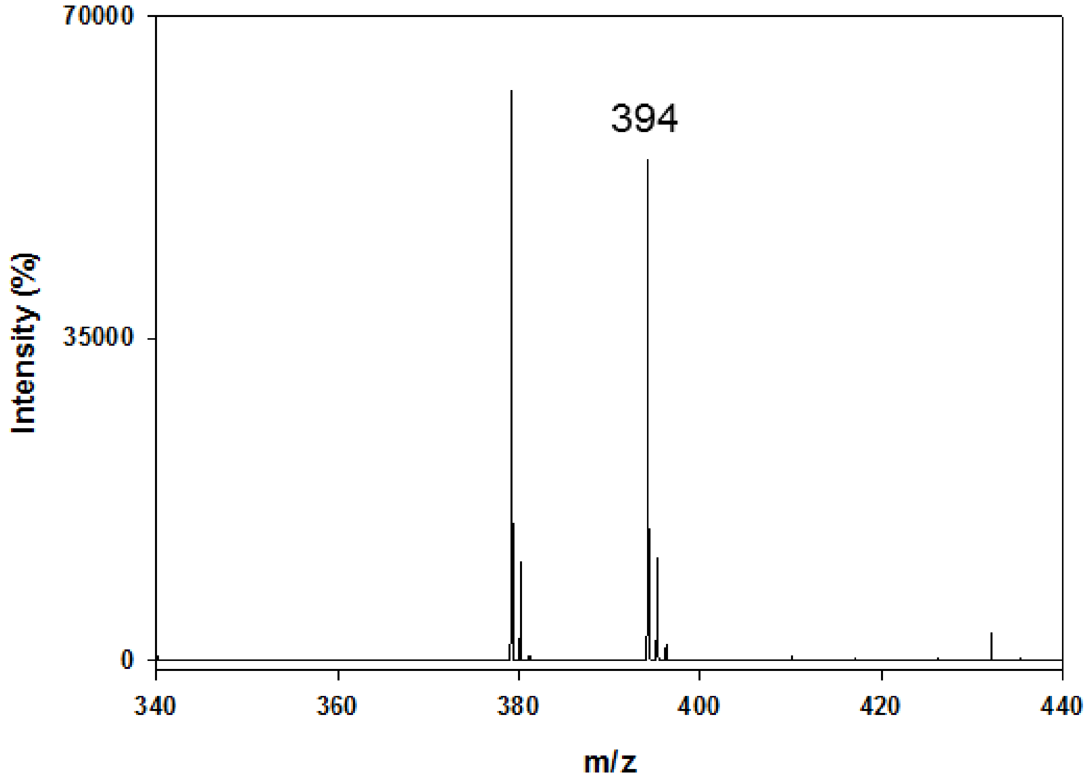

Figure S8-10 Mass spectrum for the analysis of loxoprofen spiked in human plasma, $[M+H]^+$ signal appears at m/z 394.

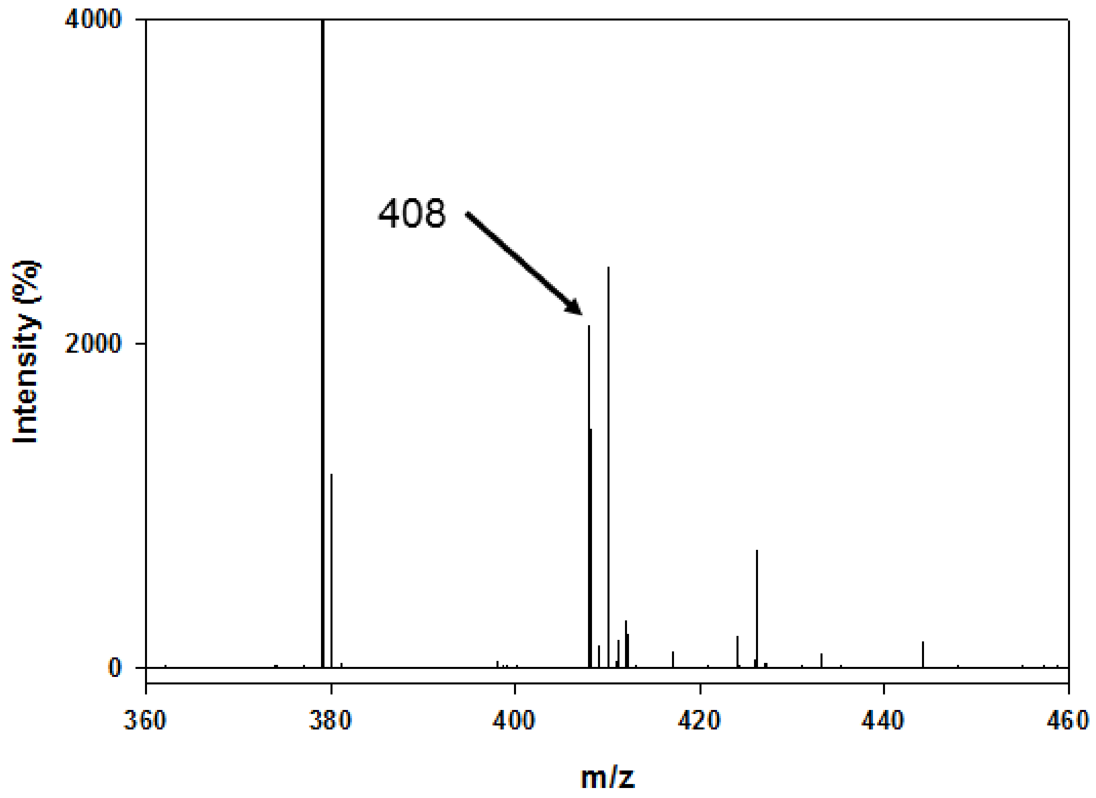

Figure S8-11 Mass spectrum for the analysis of tiaprofenic acid spiked in human plasma, $[M+H]^+$ signal appears at m/z 408.

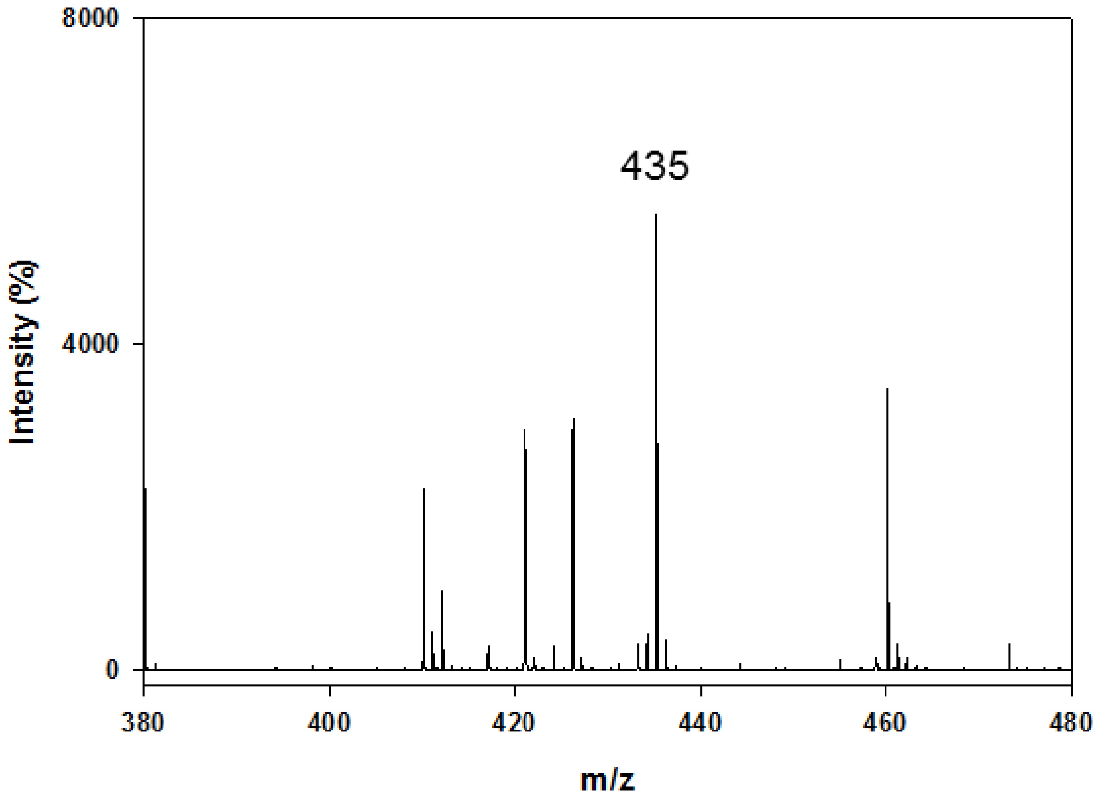

Figure S8-12 Mass spectrum for the analysis of etodolac spiked in human plasma, $[M+H]^+$ signal appears at m/z 435.

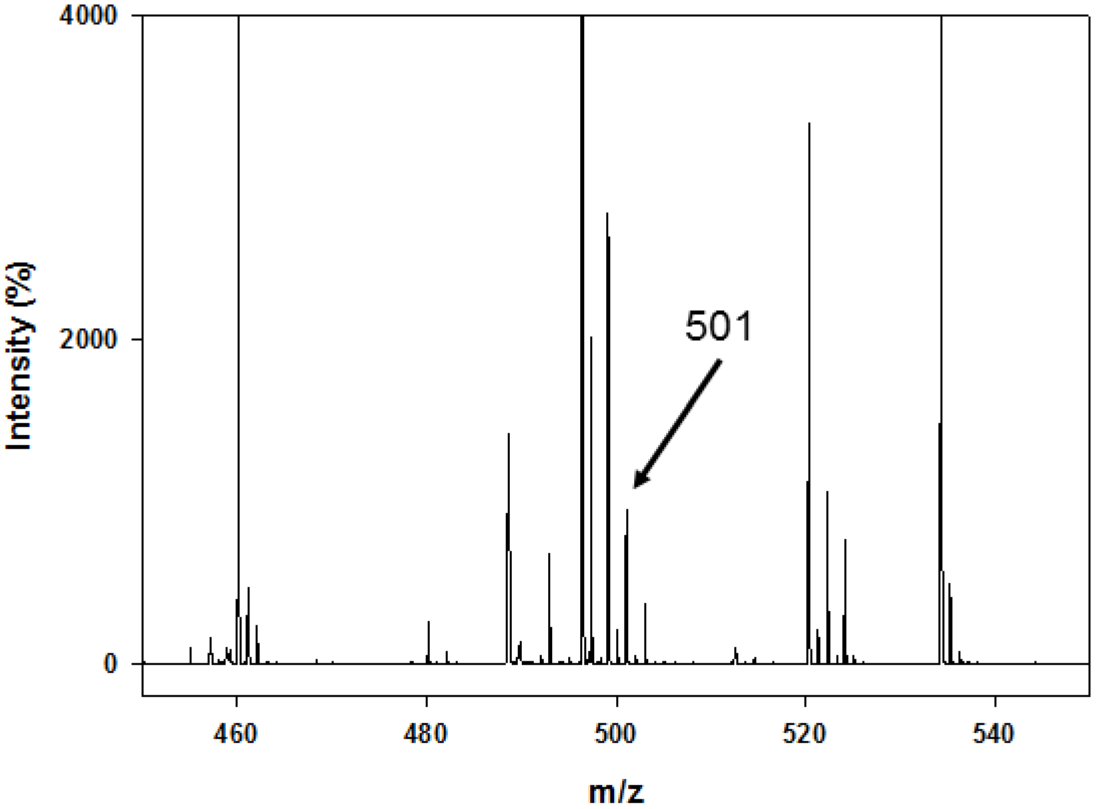

Figure S8-13 Mass spectrum for the analysis of aceclofenac spiked in human plasma, $[M+H]^+$ signal appears at m/z 501.

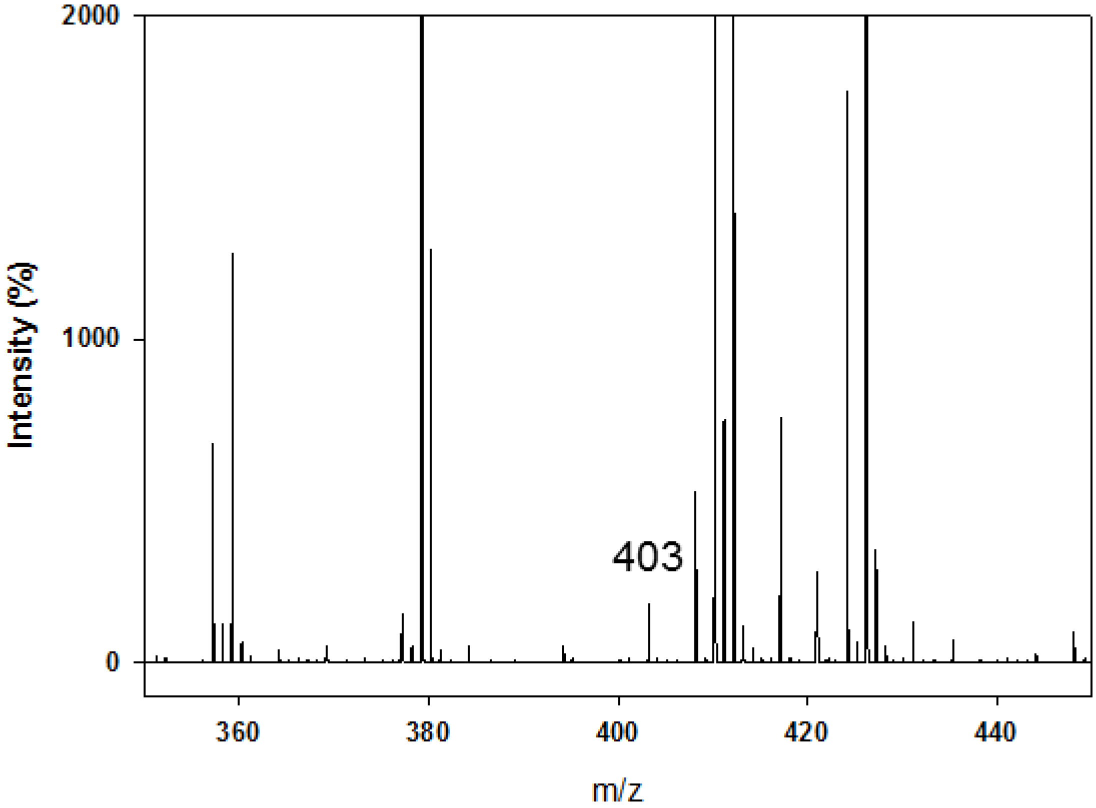

Figure S8-14 Mass spectrum for the analysis of ketorolac spiked in human plasma, $[M+H]^+$ signal appears at m/z 403.

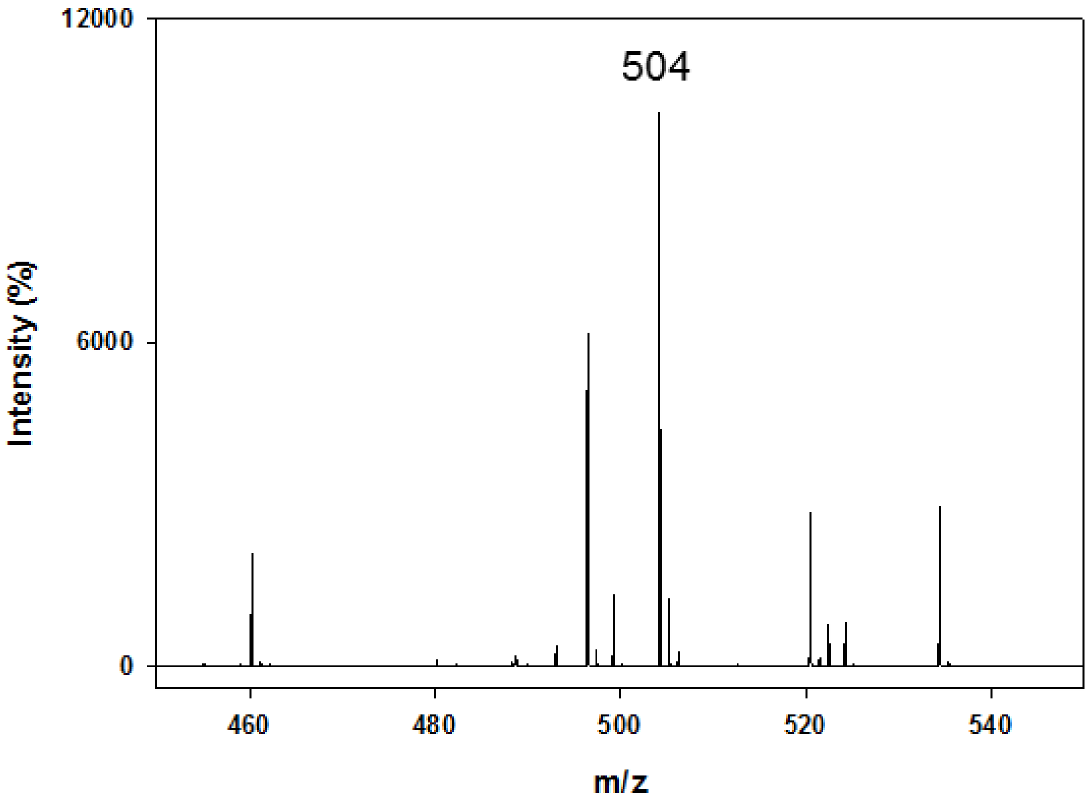

Figure S8-15 Mass spectrum for the analysis of sulindac spiked in human plasma, $[M+H]^+$ signal appears at m/z 504.

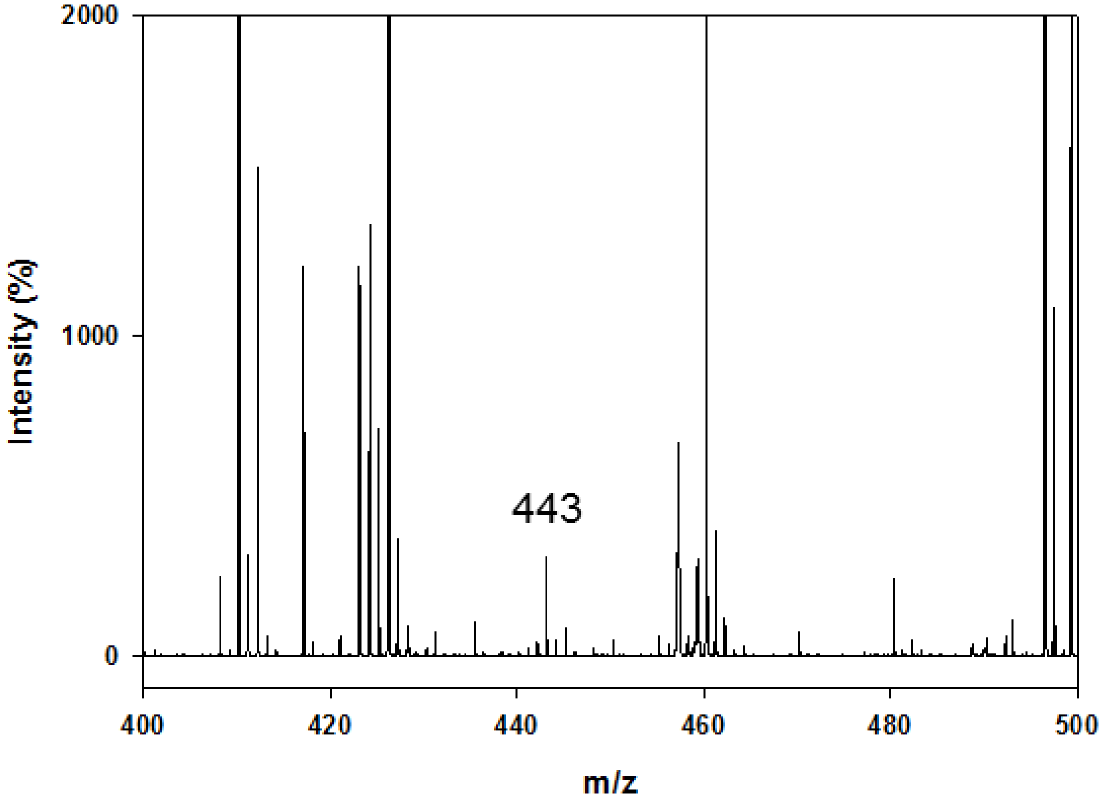

Figure S8-16 Mass spectrum for the analysis of diclofenac spiked in human plasma, $[M+H]^+$ signal appears at m/z 443.

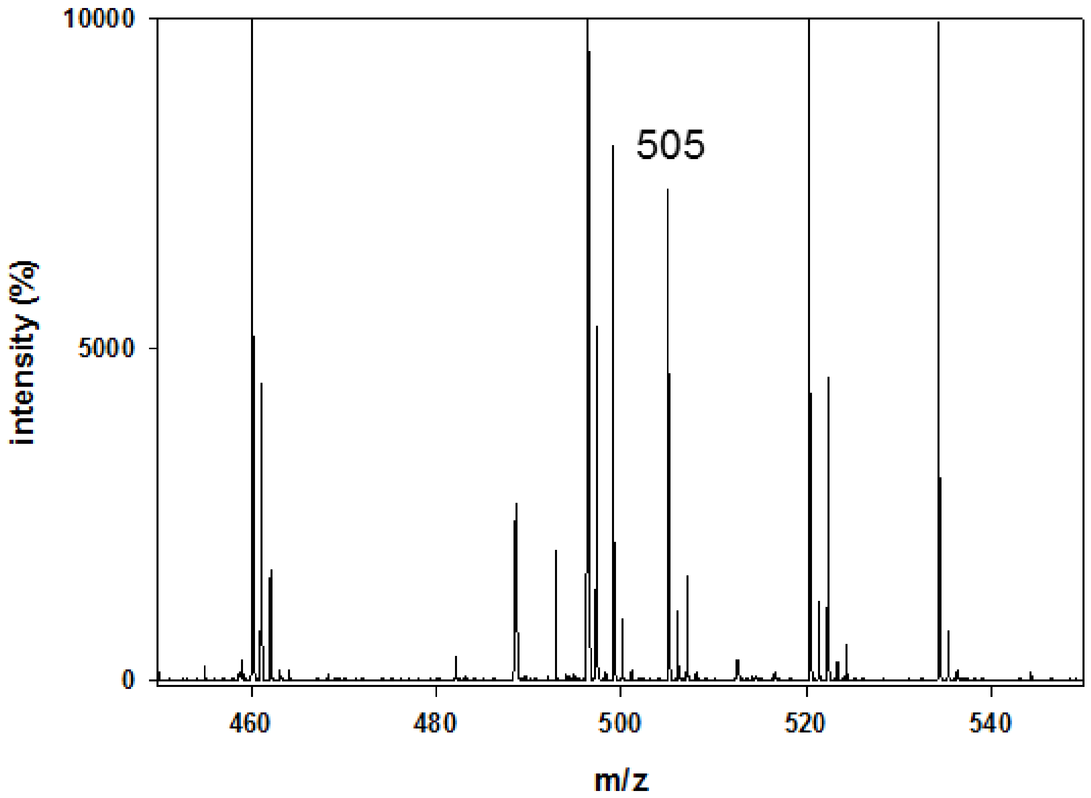

Figure S8-17 Mass spectrum for the analysis of indometacin spiked in human plasma, $[M+H]^+$ signal appears at m/z 505.

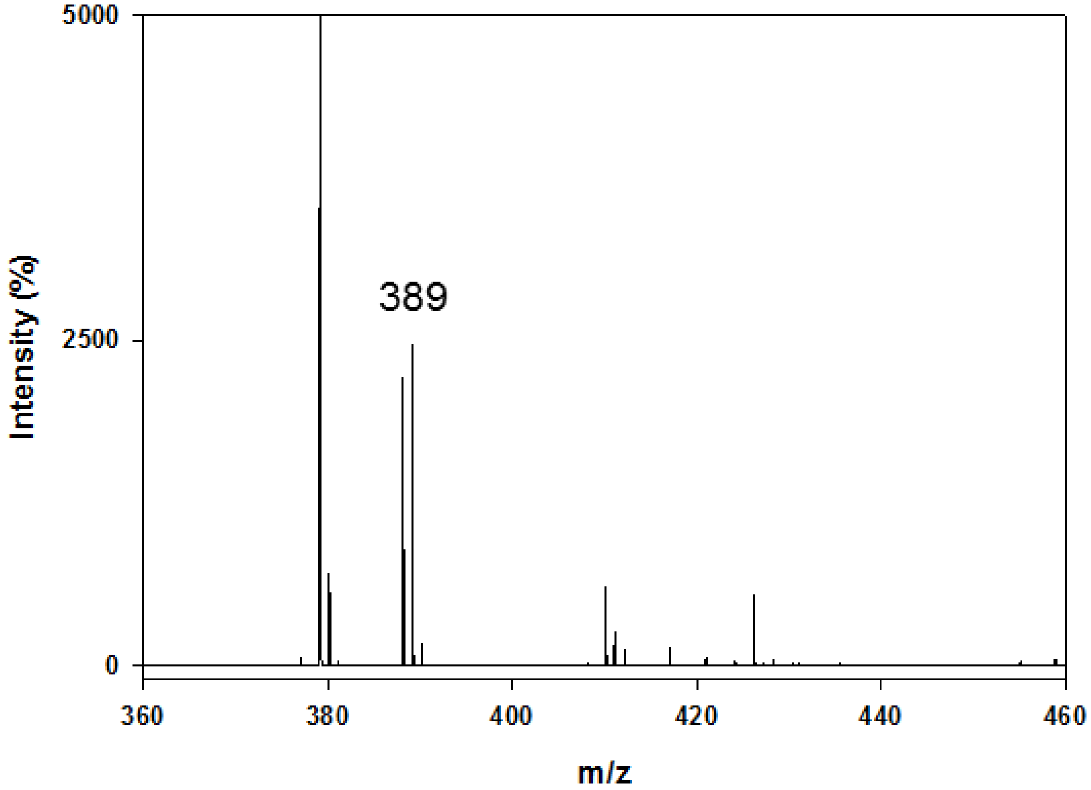

Figure S8-18 Mass spectrum for the analysis of mefenamic acid spiked in human plasma, $[M+H]^+$ signal appears at m/z 389.

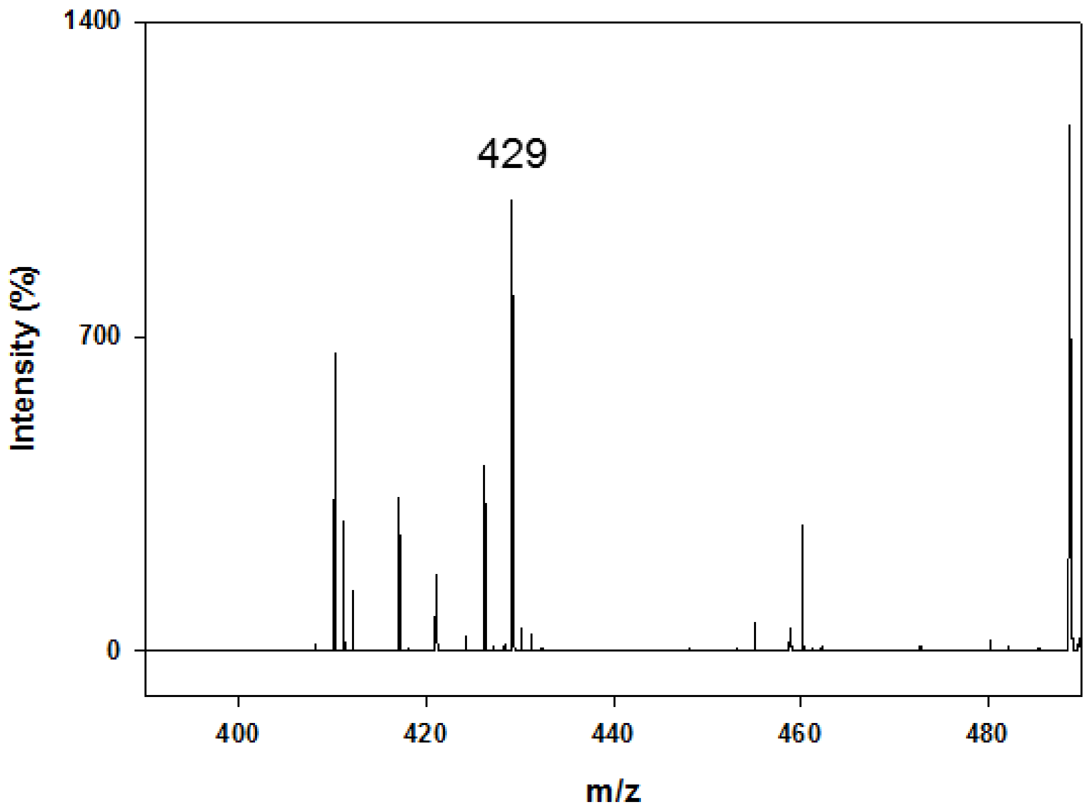

Figure S8-19 Mass spectrum for the analysis of flufenamic acid spiked in human plasma, $[M+H]^+$ signal appears at m/z 429.
